# Coronary artery bifurcation haemodynamics - comparison between phase contrast MRI and computational fluid dynamics

**DOI:** 10.1186/1532-429X-16-S1-P224

**Published:** 2014-01-16

**Authors:** Susann Beier, John Ormiston, Mark Webster, John Cater, Pau Medrano-Gracia, Alistair Young, Brett R Cowan

**Affiliations:** 1 Auckland MRI Research Group, University of Auckland, Auckland, New Zealand; 2 Department of Medicine, Auckland University, Auckland, New Zealand; 3 Department of Engineering Science, University of Auckland, Auckland, New Zealand

## Background

Coronary atherosclerosis is common at vessel bifurcations. A quantitative approach to measuring blood velocity, vorticity and more complex flow features at bifurcations would enhance the understanding of the mechanisms of atheroma development, and potentially predict vessels at highest risk. The aim of this work was to validate 4D phase contrast (PC) magnetic resonance imaging flow measurements using a simplified arterial model of the left main coronary bifurcation against computational fluid dynamic (CFD) modelling.

## Methods

**Figure 1:**
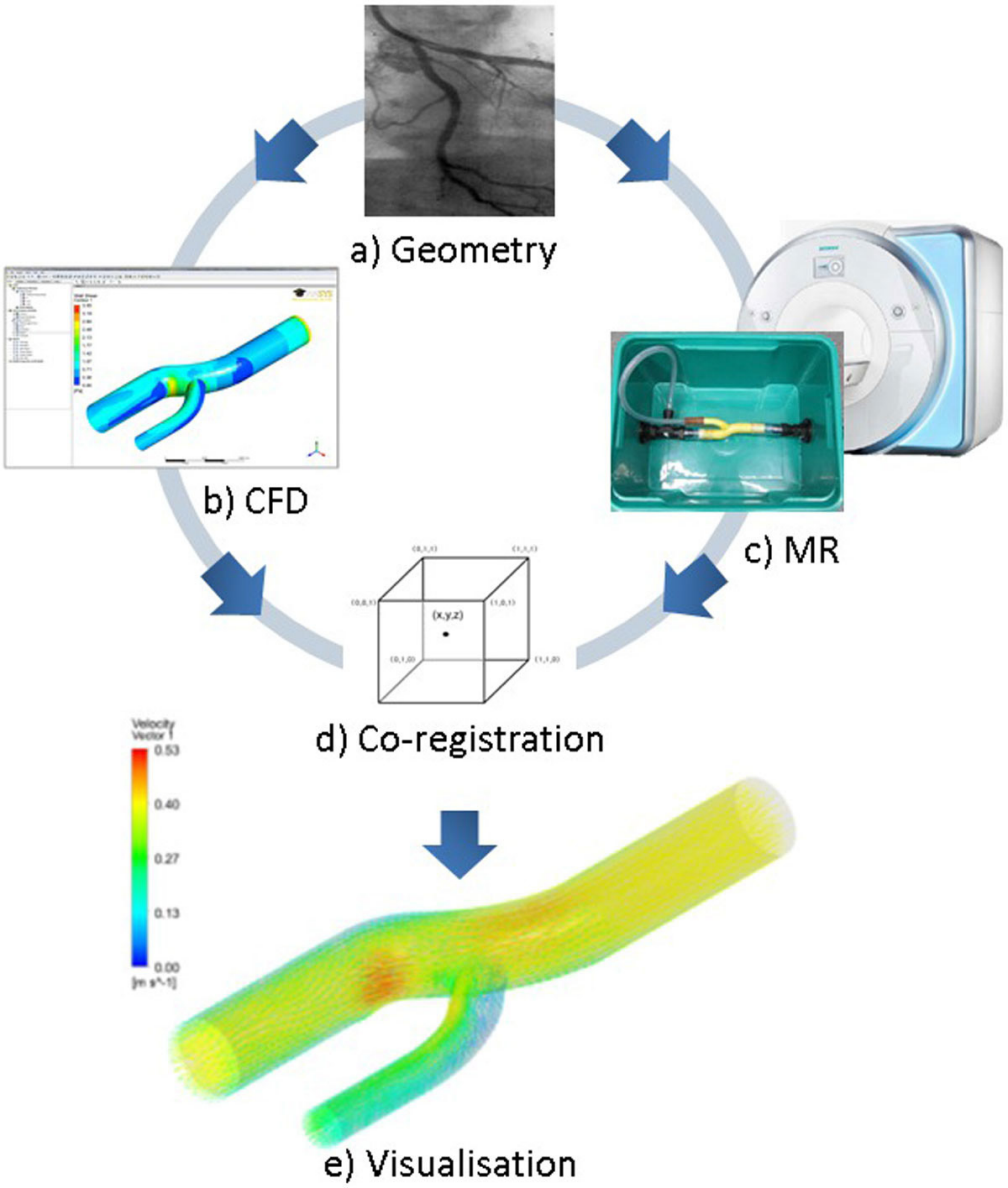
**a) Simplified left main bifurcation geometry created from CT angiography data, b) CFD simulation, c) 4D PC MR flow measurements, d) co-registration, and e) visualisation and numerical comparison of the combined results**.

A simplified left main to left anterior descending/circumflex bifurcation phantom was created from CT angiography data using 3D rapid prototyping. This was scaled up six-fold using the laws of dimensional scaling to an inlet diameter of 25 mm and connected to a pump to create a steady flow circuit in the MR scanner. Flow was measured using 4D PC MRI on a 3T Siemens Skyra (Erlangen, Germany). Custom software (MATLAB) was developed to convert the PC grey scale images to a structured data set with spacial, 3D velocity vector and time properties. The same geometry and conditions were then digitally modelled using CFD (ANSYS CFX 13.0 and Dell T5500, 64-bit 2.13 GHz six-core Intel Xeon with 12GB RAM) and the results exported to the same structured data set for comparison. The data was registered using a least squares 3D affine transformation and tri-linearly interpolated to create a co-registered 4D volume with more than 90 million data points which was then visualised as shown in Figure 1.

## Results

The standard deviation of the differences between the PC and CFD data was less than 10%. Regional flow features such as vortices were identified in the PC data, and were found to be subjectively similar to the corresponding features in the CFD data. Regions of PC artefact were identified using the CFD results, and were associated with lower spatial resolution and background eddy current effects.

## Conclusions

PC MR flow measurements show good quantitative and structural flow similarity to CFD. CFD may be useful for the identification of regions of artefact in PC data and potentially provide a means of correction or regularisation.

## Funding

Auckland Heart Group Charitable Trust.

